# Estimated Cost-effectiveness of Solar-Powered Oxygen Delivery for Pneumonia in Young Children in Low-Resource Settings

**DOI:** 10.1001/jamanetworkopen.2021.14686

**Published:** 2021-06-24

**Authors:** Yiming Huang, Qaasim Mian, Nicholas Conradi, Robert O. Opoka, Andrea L. Conroy, Sophie Namasopo, Michael T. Hawkes

**Affiliations:** 1 University of Alberta, Edmonton, Canada; 2 Department of Pediatrics, University of Alberta, Edmonton, Canada; 3 Department of Paediatrics and Child Health, Mulago Hospital and Makerere University, Kampala, Uganda; 4 Ryan White Center for Pediatric Infectious Diseases and Global Health, Indiana University School of Medicine, Indianapolis; 5 Department of Paediatrics, Kabale District Hospital, Kabale, Uganda; 6 Department of Medical Microbiology and Immunology, University of Alberta, Edmonton, Canada; 7 University of Alberta School of Public Health, Edmonton, Canada

## Abstract

**Importance:**

Pneumonia is the leading cause of childhood mortality worldwide. Severe pneumonia associated with hypoxemia requires oxygen therapy; however, access remains unreliable in low- and middle-income countries. Solar-powered oxygen delivery (solar-powered O_2_) has been shown to be a safe and effective technology for delivering medical oxygen. Examining the cost-effectiveness of this innovation is critical for guiding implementation in low-resource settings.

**Objective:**

To determine the cost-effectiveness of solar-powered O_2_ for treating children in low-resource settings with severe pneumonia who require oxygen therapy.

**Design, Setting, and Participants:**

An economic evaluation study of solar-powered O_2_ was conducted from January 12, 2020, to February 27, 2021, in compliance with the World Health Organization Choosing Interventions That Are Cost-Effective (WHO-CHOICE) guidelines. Using existing literature, plausible ranges for component costs of solar-powered O_2_ were determined in order to calculate the expected total cost of implementation. The costs of implementing solar-powered O_2_ at a single health facility in low- and middle-income countries was analyzed for pediatric patients younger than 5 years who required supplemental oxygen.

**Exposures:**

Treatment with solar-powered O_2_.

**Main Outcomes and Measures:**

The incremental cost-effectiveness ratio (ICER) of solar-powered O_2_ was calculated as the additional cost per disability-adjusted life-year (DALY) saved. Sensitivity of the ICER to uncertainties of input parameters was assessed through univariate and probabilistic sensitivity analyses.

**Results:**

The ICER of solar-powered O_2_ was estimated to be $20 (US dollars) per DALY saved (95% CI, $2.83-$206) relative to the null case (no oxygen). Costs of solar-powered O_2_ were alternatively quantified as $26 per patient treated and $542 per life saved. Univariate sensitivity analysis found that the ICER was most sensitive to the volume of pediatric pneumonia admissions and the case fatality rate. The ICER was insensitive to component costs of solar-powered O_2_ systems. In secondary analyses, solar-powered O_2_ was cost-effective relative to grid-powered concentrators (ICER $140 per DALY saved) and cost-saving relative to fuel generator-powered concentrators (cost saving of $7120).

**Conclusions and Relevance:**

The results of this economic evaluation suggest that solar-powered O_2_ is a cost-effective solution for treating hypoxemia in young children in low- and middle-income countries, relative to no oxygen. Future implementation should prioritize sites with high rates of pediatric pneumonia admissions and mortality. This study provides economic support for expansion of solar-powered O_2_ and further assessment of its efficacy and mortality benefit.

## Key Points

**Question:**

Is solar-powered oxygen delivery (solar-powered O_2_) a cost-effective intervention for use in children younger than 5 years with hypoxemia in low-resource settings?

**Findings:**

This economic evaluation compared the costs and health outcomes of solar-powered O_2_ with (1) null case with no oxygen, (2) grid-powered oxygen concentrators, and (3) fuel generator-powered concentrators. Use of solar-powered O_2_ was cost-effective relative to the null case and grid-powered concentrators and was cost-saving relative to fuel generator-powered concentrators.

**Meaning:**

The results of this economic evaluation suggest that solar-powered O_2_ is a cost-effective intervention for pediatric patients with hypoxemia in low-resource settings.

## Teaser

This economic evaluation assesses whether solar-powered oxygen delivery is a cost-effective intervention for children younger than 5 years with hypoxemia in low-resource settings.

## Introduction

Hypoxemia is present in 10% to 15% of children admitted to hospitals globally.^1^ Pneumonia, the leading cause of childhood mortality outside the neonatal period, is a common cause of hypoxemia.^2,3^ Based on a meta-analysis of 13 studies involving 13 928 children with pneumonia, hypoxemia is a strong predictor of mortality, increasing the risk of dying 5-fold.^4^ Although bacterial pneumonia is the leading cause of hypoxemia, other pathogenic and congenital pathologies may also lead to hypoxemia as a final common pathway to respiratory failure.^5^ Regardless of etiology, hypoxemia requires treatment with supplemental oxygen. Improved oxygen systems reduce pneumonia mortality by an estimated 35%, but access remains unreliable in low- and middle-income countries (LMICs).^6^ Given that pneumonia is responsible for approximately 900 000 childhood deaths annually, access to oxygen is an important public health issue.^7,8^

Although oxygen is included on the World Health Organization (WHO) list of essential medicines,^9^ it may not be available in hospitals and health centers in LMICs because of cost and/or logistical challenges.^10,11^ During the current COVID-19 pandemic, oxygen needs globally and in low-resource settings are expected to increase, exacerbating the gap in availability. Methods currently used in low-resource settings include compressed oxygen cylinders and grid-powered oxygen concentrators.^12,13^ Cylinders require supply chains linking oxygen production plants to hospitals, which may be compromised by poor road conditions, costs of transportation, and weak supply chain management.^12,13^ Oxygen losses due to leakage can also affect the cost-effectiveness and reliability of oxygen cylinders.^14,15^ Oxygen concentrators, though shown to be more cost-effective and user-friendly than cylinders, depend on a reliable and uninterrupted supply of electricity, which is often unavailable in resource-constrained settings.^16^ A previous systematic review showed that 26% of health facilities in sub-Saharan Africa reported no access to electricity, and only 28% of centers reported reliable access.^17^ Power outages lasted a median of 7% of the time monitored in a study from western Kenya (range, 1%-58%).^16^ In that study, facilities experienced a median of 7 power outages per week (interquartile range, 7-16 outages) lasting a median of 17 minutes each (interquartile range, 11-27 minutes).^16^

Solar-powered oxygen delivery (solar-powered O_2_) has been shown to be an effective solution for supplemental oxygen delivery in low-resource settings.^18,19^ Solar-powered oxygen delivery has been described in detail previously and implemented at 2 hospitals in Uganda to successfully treat children with hypoxemia.^18,19^ In brief, photovoltaic cells installed on the roofs of hospitals collect solar energy, which is stored as electricity in a battery bank, then used to power an oxygen concentrator for production of medical-grade oxygen.^18^ The efficacy of solar-powered O_2_ was demonstrated in a proof-of-concept pilot study and a randomized clinical trial that showed clinical noninferiority compared with cylinder oxygen.^18,19^ Solar-powered oxygen delivery has several advantages, including low operating costs, consistency and reliability through grid-power outages, being user-friendly for hospital staff, reduced oxygen waste, and reduced carbon footprint owing to exclusive use of freely available inputs of solar energy and air.^18,19^

Having demonstrated that solar-powered O_2_ is a feasible, safe, and effective solution to the oxygen gap in LMICs,^18,19^ we now seek to answer whether solar-powered O_2_ is a cost-effective intervention for treating pediatric patients with hypoxemia in low-resource settings. We followed the WHO Choosing Interventions That Are Cost-Effective (WHO-CHOICE) methodology and the associated guidelines for performing a generalized cost-effectiveness analysis.^20^ One of the main benefits of this approach is the use of a “null” case, wherein the effects of all currently available interventions are removed, allowing for more effective comparison between different interventions.^20^ We hypothesized that solar-powered O_2_ would be cost-effective relative to the null case (no oxygen), using the gross domestic product (GDP) per capita of target LMICs as a cost-effectiveness threshold. Secondary analyses compared solar-powered O_2_ with oxygen concentrators powered by grid electricity and fuel generators. These analyses may more closely approximate the decision facing administrators and policy makers on the use of solar-powered O_2_.

## Methods

### Cost-effectiveness Analysis

This economic evaluation was completed from January 12, 2020, to February 27, 2021. The decision analytic framework used was a cost-effectiveness comparison between 2 scenarios: the intervention (solar-powered O_2_) and a comparator condition. For the primary analysis, the comparator condition was the null case (no oxygen); for secondary analyses, the comparator conditions were grid-powered concentrators or fuel generator-powered concentrators.

The setting for implementation of solar-powered O_2_ was a single rural or remote health facility with inpatient pediatric services in an LMIC without prior available medical oxygen.^10^ Cost-effectiveness of solar-powered O_2_ was assessed from health care sector and societal perspectives.^21,22^ A time horizon of 10 years was used. We followed the Consolidated Health Economic Evaluation Reporting Standards (CHEERS) guideline in reporting our findings (eAppendix and eMethods in the Supplement). Ethics approval was granted by the Health Research Ethics Board at the University of Alberta. The cost-effectiveness analysis used parameters that were derived from the literature and past experience installing the systems. There were no patient-specific data here; therefore, patient consent was not required or relevant.

### Health Outcomes and Costs

The published literature was used when possible to estimate input parameters for health outcomes and costs (Table 1; eTable 1 in the Supplement).^23,24,26,31,32,34^ When published data were not available, we used data from our own experience implementing and evaluating solar-powered O_2_ in Uganda (Table 1).^18,19^

**Table 1.** Parameter Estimates for Cost-effectiveness of Solar-Powered O_2_ Systems and Direct Medical and Nonmedical Costs Associated With Hospitalization for Hypoxemia

Parameter	Base (range)^a^	Distribution	Reference
**Factors for calculation of DALY**			
Annual No. of childhood pneumonia admissions (single health facility)	431 (82-987)	Poisson	Nabwire et al,^10^ 2018
Proportion of patients admitted with pneumonia who are hypoxemic	0.133 (0.093-0.375)	Beta	Subhi et al,^1^ 2009
Ratio of total hypoxemia cases: hypoxemic pneumonia cases	1:0.66 (1:0.33-1:1.1)	Beta	McCollum et al,^23^ 2013
Hypoxemic pneumonia case fatality rate (with oxygen)	0.089 (0.034-0.153)	Beta	Lazzerini et al,^4^ 2015
Relative risk reduction of mortality with oxygen	0.35 (0.22-0.48)	Beta	Duke et al,^6^ 2008
Age of patient, y	1.7 (0.0-5.0)	Gamma	Usen et al,^24^ 1999
Life expectancy, y	59.2 (52.0-80.0)	Gamma	World Bank^25^
Time on oxygen, d			
Survivors	4.00 (1.00-8.00)	Gamma	Nantanda et al,^26^ 2014
Fatal cases	1.80 (0.14-15.00)	Gamma	Hawkes et al,^19^ 2018
**Direct medical costs**			
Solar-powered oxygen system: photovoltaic cells (panels), batteries, and wiring			
Hours of available sunlight	5 (3-8)	Gamma	Hawkes et al,^19^ 2018
Price of solar panels, $/W	2.92 (1.93-3.73)	Gamma	Turnbull et al,^18^ 2016; Fu et al,^27^ 2017
Price of inverter	1132 (566-1698)	Gamma	Hawkes et al,^19^ 2018
Price of charge controller	1581 (790-2371)	Gamma	Hawkes et al,^19^ 2018
Required duration of backup battery supply	48 (24-72)	Gamma	Hawkes et al,^19^ 2018
Price of batteries, $/Ah	1.73 (0.61-3.47)	Gamma	Turnbull et al,^18^ 2016; Rahman et al,^28^ 2018
Life span of batteries, y	5 (2-8)	Gamma	Turnbull et al,^18^ 2016
Price of wiring and shelving	1383 (691-2074)	Gamma	Hawkes et al,^19^ 2018
Price of labor and travel for installation	1418 (709-2127)	Gamma	Hawkes et al,^19^ 2018
Life span of solar-powered O_2_ system, y	10 (5-20)	Gamma	Turnbull et al,^18^ 2016; World Health Organization^20^
Oxygen concentrator			
Price of oxygen concentrator, $	1026 (615-1352)	Gamma	Bradley et al,^29^ 2015; Turnbull et al,^18^ 2016; Hawkes et al,^19^ 2018
Oxygen concentrator power consumption, kW	0.28 (0.23-0.33)	Gamma	Turnbull et al,^18^ 2016; Hawkes et al,^19^ 2018
Life span of oxygen concentrator, y	7 (2-10)	Gamma	Bradley et al,^29^ 2015
Annual maintenance cost of oxygen concentrator, $	669 (197-860)	Gamma	Bradley et al,^29^ 2015; Turnbull et al,^18^ 2016; Hawkes et al,^19^ 2018
Other direct medical costs			
Cost of hospitalization for pneumonia, $/patient	203 (152-255)	Gamma	Edejer et al,^30^ 2005
**Nonmedical costs**			
Duration of admission, d			
Survivors	4 (1-8)	Gamma	Nantanda et al,^26^ 2014
Fatal cases	1.80 (0.14-15.00)	Gamma	Hawkes et al,^19^ 2018
Daily household income, $	1.61 (1.06-2.10)	Gamma	Uganda Bureau of Statistics,^39^
Distance traveled for treatment, km	11.2 (5-80)	Gamma	Peterson et al,^31^ 2004; Graham et al,^5^ 2018; Idro and Aloyo^32^ 2004
Cost of transportation, $/km	0.31 (0-1.04)	Gamma	Sadigh et al,^33^ 2016; Matovu et al,^34^ 2014
Daily expenses (includes meals and accommodation for caregiver), $	2.99 (2.34-8.82)	Gamma	Sadigh et al,^33^ 2016; Anderson et al,^35^ 2017

Abbreviations: Ah, ampere hour; DALY, disability-adjusted life-year; solar-powered O_2_, solar-powered oxygen delivery.

^a^ All nominal costs adjusted to real costs in 2019 in US dollars.

The GDP deflator method derived from method 2 by Turner and colleagues^36^ was used to adjust for inflation and convert costs to a single base year (2019). We used 2019 as the base year because the most recent GDP deflator statistics were available up to 2019.^36^ The GDP deflator for a given period reflects the average annual rate of inflation in the economy as a whole during that period. Gross domestic product deflators are available from the World Bank.^37^ Local costs were adjusted using local inflation rates before converting to US dollars.^36^ For conversion of local currency to US dollars, we used historical conversion rates.^38^ The real costs of solar-powered O_2_ components, consumables, and equipment for alternative oxygen delivery methods are shown in Table 1 and eTable 1 in the Supplement.

With respect to nonmedical costs, we included opportunity costs and direct costs. Opportunity costs were the wages for 1 caregiver for the duration of the hospitalization and were based on the household income in Uganda.^39^ Direct costs included travel, accommodation, and food for 1 caregiver for the duration of the hospitalization. Food cost was calculated as the difference between the daily cost of purchasing food and the cost of food in the home environment if the child was not hospitalized.^33,35^

The outcome (health effect) of interest was the number of disability-adjusted life-years (DALYs) saved with solar-powered O_2_. The DALYs represent a widely used public health metric of disease burden. The WHO advocates the use of DALYs for generalized cost-effectiveness analyses and recommends this methodology for comparability.^20^ The DALYs lost due to a disease refers to the combination of years of life lost (YLL) due to premature mortality and years of life lost due to disability (YLD), which accounts for the loss of health by applying a disability weighting. In the context of this study, we focused on YLL, under the assumption that otherwise healthy children who recover from pneumonia will not have long-standing disability. In the case of fatal childhood pneumonia, YLL were calculated as the difference between the life expectancy for patients (based on vital statistics) and the age at death.

All DALYs were calculated using the following formulas:

*DALY* = *YLL* + *YLD* ≈ *YLL* and

*YLL* = *number of deaths* × *standard life expectancy at age of death*.

For the DALY calculation, we neglected the YLD, such that YLL accounted for all the DALYs lost. This was based on the assumption that children who recover from pneumonia do not have residual morbidity.^40,41^

For our base case scenario, both health outcomes and costs were discounted at 3% following the WHO-CHOICE recommendations.^20^ Discounting was performed using a discounting factor (DF) given by the following formula^20^:


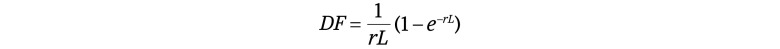
.

### Calculation of Cost-Effectiveness

The comparison between the 2 scenarios used the incremental cost-effectiveness ratio (ICER) to assess the trade-off between improved health outcomes and increased costs. The ICER was defined as the difference in cost between interventions, divided by the difference in their effect (DALYs saved):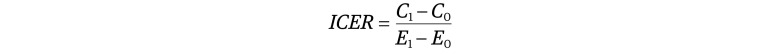.The threshold for cost-effectiveness was assumed to be the GDP per capita in representative LMICs.^42^ We used the GDP per capita of Uganda, where solar-powered O_2_ was pioneered, and the lowest GDP per capita in the world (South Sudan, GDP of $220) for maximum stringency.

### Statistical Analysis

To evaluate the association of uncertainty with cost-effectiveness, we conducted univariate sensitivity analyses in which a single key input parameter was varied throughout the plausible range while maintaining other parameters at their base case values. The resulting variation in the ICER was displayed as a tornado plot (eMethods in the Supplement). Additionally, a probabilistic sensitivity analysis was performed. Input parameters were randomly sampled from their assumed probability distributions (Table 1) to assess stability of the calculated ICER when multiple input parameters were varied simultaneously. The resulting incremental costs, incremental health outcomes (DALYs saved), and ICERs were plotted on a cost-effectiveness plane and used to generate a cost-effectiveness acceptability curve. Further details are provided in the eMethods in the Supplement.

We used bootstrap analysis to sample the costs and health outcomes concurrently, using the probability distributions of the input variables. We generated multiple estimates of the ICER and its component variables, and we used these to calculate the 95% CI for each variable (2.5th percentile and 97.5th percentile). Analyses were performed using R statistical software, version 3.6.2 (R Core Team).

## Results

### Direct Medical Costs of Solar-Powered O_2_

Under the base case assumptions, installation of a solar-powered O_2_ system at a single hospital required a capital cost of $12 411. This cost comprised photovoltaic cells ($3930, at $2.92/W)^27^, batteries ($1941, at $1.73/ampere hour)^28^, an oxygen concentrator ($1026)^29^, and additional components and setup costs ($5513). Ongoing costs were estimated at $10 528 over 10 years for maintenance ($5776), battery replacement ($3108), and concentrator replacements ($1644). Thus, the total incremental cost of solar-powered O_2_ relative to the null case without oxygen over the expected 10-year life span of the solar-powered O_2_ system was $22 939 (Table 2). Based on lifetime costs and the number of patients treated (Table 2), the cost of solar-powered O_2_ is $26 per patient treated (ie, $22 939 per 869 patients).

**Table 2.** Health Outcomes and Costs With and Without Solar-Powered O_2_ at a Single Health Facility Over 10 Years

Parameter	No solar-powered O_2_ (95% CI)	With solar-powered O_2_ (95% CI)	Prevented by solar-powered O_2_ (95% CI)	Difference, % (95% CI)
**Events**				
Hospitalizations with hypoxemia	869 (78 to 3580)	869 (78 to 3580)	0	0
Deaths	119 (9 to 559)	77 (6 to 352)	42 (3 to 205)	35 (23 to 48)
DALYs	20 535 (2434 to 127 893)	21 675 (2586 to 134 520)	1140 (106 to 8541)	6 (2 to 14)
**Costs, $**				
Direct medical costs				
Solar-powered O_2_ (capital and maintenance)	0	22 939 (15 034 to 33 999)	22 939 (15 034 to 33 999)	NA
Antibiotics and other treatment	138 407 (11 650 to 518 564)	138 407 (11 650 to 518 564)	0	0
Total medical costs	138 407 (11 650 to 518 564)	161 346 (31 913 to 543 164)	22 939 (15 034 to 33 999)	17 (4 to 180)
Nonmedical costs				
Loss of earnings by caregiver	4476 (269 to 19 467)	4603 (278 to 20 246)	128 (−432 to 964)	3 (−11 to 12)
Other direct nonmedical	13 453 (505 to 71 536)	13 689 (523 to 73 553)	236 (−828 to 1870)	2 (−8 to 10)
Total nonmedical costs	17 929 (934 to 90 166)	18 293 (954 to 91 594)	364 (−1308 to 2737)	2 (−9 to 10)
Total cost	156 336 (13 349 to 586 920)	179 639 (33 669 to 614 795)	23 303 (14 999 to 34 457)	15 (4 to 160)

Abbreviations: DALY, disability-adjusted life-year; NA, not available; solar-powered O_2_, solar-powered oxygen delivery.

### Nonmedical Costs of Solar-Powered O_2_

The societal perspective adds the expected costs incurred by the families of patients (Table 2). One hospital admission is expected to cost a family approximately $6.94 in transportation costs and $4.60 for each day of hospitalization in direct and opportunity costs, adding $18 293 to the cost of treating patients with hypoxemia over the 10-year project horizon (10% of total cost).

### Health Outcomes and ICER

For a hospital with 431 pneumonia admissions per year, the system could treat 869 hypoxemic patients over 10 years (see Table 1 for assumed input parameters):
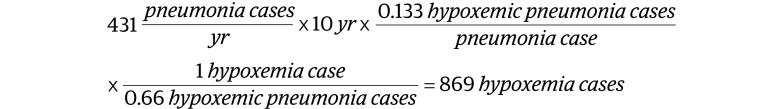
.Assuming a mortality reduction of 35% with oxygen, the solar-powered O_2_ system would be expected to save 42 lives and 1140 DALYs, relative to the null case (Table 2):
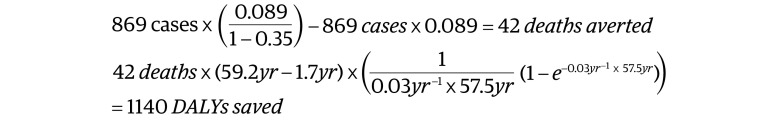
.The incremental cost of solar-powered O_2_ was therefore $542 per life saved (ie, $22 939 per 42 lives saved). The ICER was $20 per DALY saved (95% CI, $2.83-$206). Using the GDP per capita of Uganda ($604) as a threshold for cost-effectiveness, solar-powered O_2_ was highly cost-effective.

### Sensitivity Analysis

**Figure 1.**
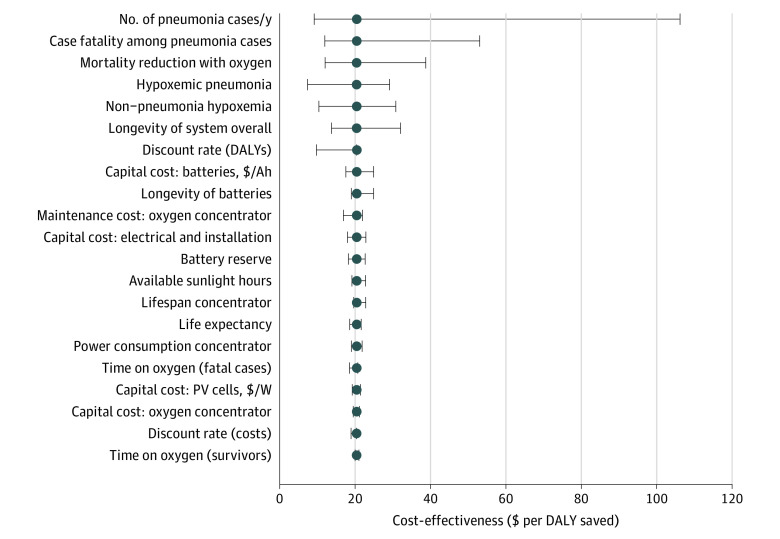
One-Way Sensitivity Analysis of the Incremental Cost-Effectiveness Ratio (ICER) Estimate for Solar-Powered Oxygen Delivery Relative to Null Case (No Oxygen) Values are ICER ($ per disability-adjusted life-year [DALY] saved) with whiskers representing the outcome of univariate sensitivity analyses over a plausible range of parameter inputs. Variables were ranked based on level of outcome (from top to bottom). Details of the range of input parameters are given in Table 1. Ah indicates ampere hour; PV, photovoltaic.

We examined the sensitivity of our ICER estimate to variations in the key input variables. The ICER estimate was most sensitive to the number of children presenting with pneumonia and the mortality rate of pneumonia (Figure 1). The effects of component costs on the ICER (unit cost of photovoltaic panels and batteries) were small.

**Figure 2.**
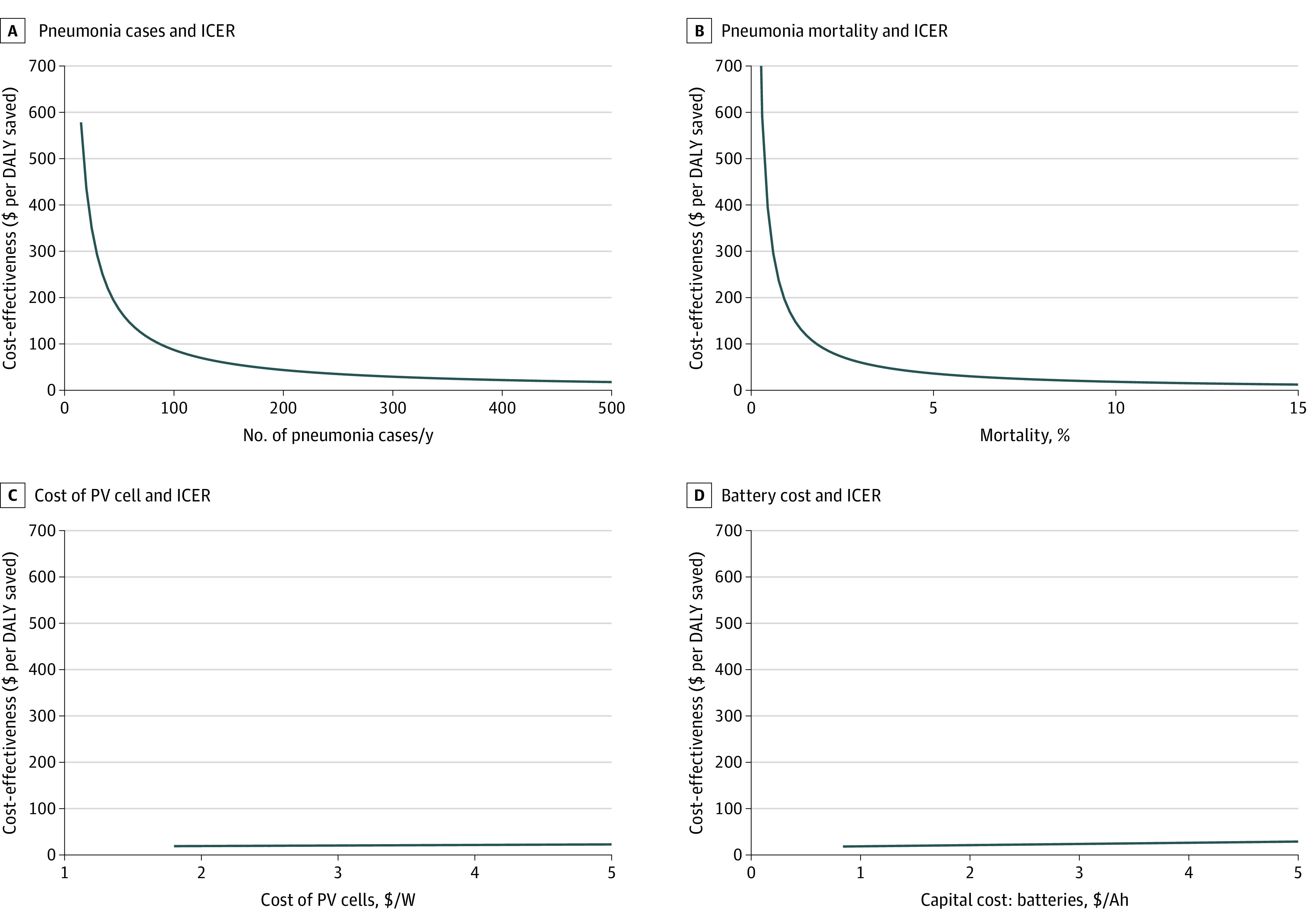
One-Way Sensitivity Analysis of Key Parameters in the Incremental Cost-Effectiveness Ratio (ICER) Estimate for Solar-Powered Oxygen Delivery (Solar-Powered O_2_) Relative to Null Case (No Oxygen) A, Nonlinear relationship between number of pneumonia cases and ICER. Solar-powered O_2_ was most cost-effective at high-volume facilities. B, Nonlinear relationship between pneumonia mortality and ICER. Due to differences in referral patterns, resources, and capacity for management, mortality in childhood pneumonia may vary between sites. Solar-powered O_2_ was most likely to be cost-effective at high mortality facilities. ICER estimate varies linearly and was relatively insensitive to uncertainties in unit costs of C, photovoltaic (PV) panels and D, batteries. Of these, a change in the unit cost for batteries had the largest effect on ICER. Ah indicates ampere hour; DALY, disability-adjusted life-year.

In a detailed 1-way sensitivity analysis for 4 selected input variables, the ICER was inversely proportional to parameters used to compute DALY saved (Figure 2A and B), including the number of children treated over the life of the system and the case fatality rate of children presenting with pneumonia. The ICER was favorable (<$604 per DALY saved) when the number of patients with pneumonia exceeded 15 per year and when the case fatality rate exceeded 0.3%. In contrast, the ICER varied linearly with component costs (Figure 2C and D) and was insensitive to changes in the component costs over a plausible range of parameter inputs.

**Figure 3.**
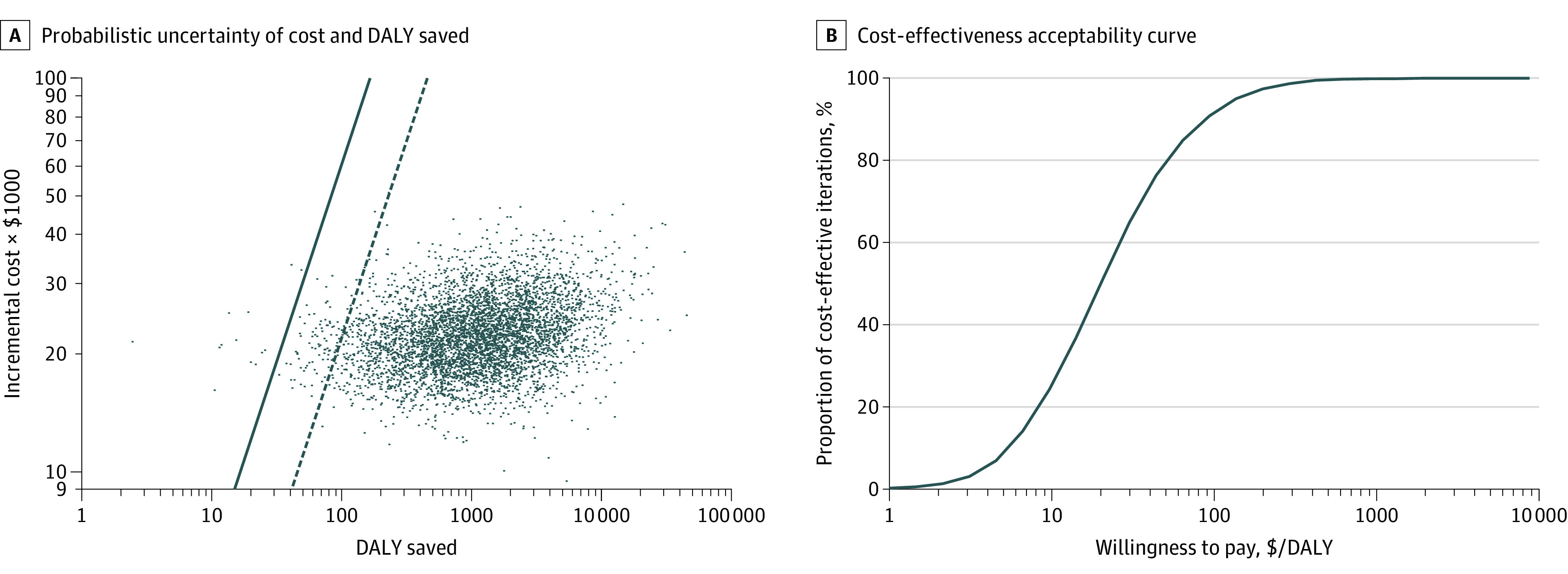
Sensitivity Analysis of Incremental Total Cost and Disability-Adjusted Life-Years (DALYs) Saved With Solar-Powered Oxygen Delivery (Solar-Powered O_2_) Relative to Null Case (No Oxygen) and Cost-effectiveness Acceptability Curve A, Scatterplot showing the probabilistic uncertainty of costs and DALYs saved through 5000 computer simulations. The solid line represents a threshold for cost-effectiveness of $604/DALY saved, corresponding to the gross domestic product (GDP) per capita of Uganda, where solar-powered O_2_ was first pioneered.^18^ The dashed line shows an alternative threshold of $220/DALY saved, the GDP per capita of South Sudan, lowest in the world. A total of 99.7% and 97.8% of simulations were cost-effective using these 2 thresholds, respectively. B, Cost-effectiveness acceptability curve suggests 95% CIs that solar-powered O_2_ will be cost-effective beyond a willingness-to-pay threshold of $136/DALY saved.

In a probabilistic multiway sensitivity analysis, the ICER was favorable (<$604 per DALY saved) in 99.7% of simulations (Figure 3A).^25^ At an alternative threshold of $220, corresponding to the lowest GDP per capita of any country globally (South Sudan), solar-powered O_2_ remained cost-effective in 97.8% of simulations.^25^ The cost-effectiveness acceptability curve (Figure 3B) showed that, at a willingness to pay of $136 per DALY saved, the likelihood of the intervention being cost-effective was 95%.

### Comparison to Other Methods of Oxygen Delivery

The direct medical cost of grid-powered oxygen concentrators over 10 years was $11 165 (eTable 2 in the Supplement). Compared with grid-powered concentrators and accounting for inconsistency of grid electricity (base case 7% power outage), solar-powered O_2_ was associated with 3 lives and 80 DALYs saved at an incremental cost of $11 190 (ICER $140 per DALY; 95% CI, $1.46-$1483) (eTable 2 in the Supplement). The ICER estimate was sensitive to the grid-power availability, increasing sharply as the grid-power failures became infrequent (eFigure 1 in the Supplement). The ICER was favorable (<$604 per DALY saved) when the proportion of time without power exceeded 1.6%. The ICER estimate varied linearly and was relatively insensitive to the price of grid electricity (eFigure 1 in the Supplement). The probabilistic multiway sensitivity analysis and cost-effectiveness acceptability curve are shown in eFigure 2 in the Supplement.

Compared with fuel generator-powered concentrators, solar-powered O_2_ did not save lives or DALYs but was associated with a cost saving of $7120 during the life of the equipment. Accounting for uncertainties in the parameters, this estimate had a wide 95% CI, ranging from a cost saving of $59 876 to an excess cost of $11 673 (eTable 3 in the Supplement).

## Discussion

In resource-limited settings, solar-powered O_2_ has been previously shown to be safe and effective and to run reliably off the grid for the treatment of young children with hypoxemia.^18,19^ The results of our analysis suggest that solar-powered O_2_ is also cost-effective relative to the null case (no oxygen), cost-effective relative to grid-powered concentrators, and cost-saving relative to fuel generator-powered concentrators.

We calculated an ICER of solar-powered O_2_ of $20 per DALY saved, relative to the null case (no oxygen). If Uganda’s GDP ($604) is used as a threshold, solar-powered O_2_ is a cost-effective investment for health facilities with no prior oxygen. In other LMICs, we expect solar-powered O_2_ to be cost-effective because the ICER was less than $220, the lowest GDP per capita globally (South Sudan), in 97.8% of simulations (Figure 3A).^25,43^ A previous study found that the ICER of cylinder oxygen (an alternative method of oxygen delivery) was $54 per DALY saved relative to the null case.^14^ Solar-powered oxygen delivery appears to be more cost-effective; however, the ICER for cylinder oxygen was well within the limits of uncertainty of our estimate for ICER of solar-powered O_2_ (95% CI, $2.83-$206), and differences in methods and assumptions between this previous study and ours could confound this comparison. This ICER can also be situated within a suite of other nonalternative childhood pneumonia interventions, such as pneumonia case management ($73 per DALY saved), pneumococcal conjugate vaccine ($100 per DALY saved), and *Haemophilus influenzae* type b vaccine ($202 per DALY saved).^30,44,45,46^ Our analysis also suggested that solar-powered O_2_ is cost-effective relative to grid-powered concentrators ($140 per DALY saved) and cost-saving relative to fuel generator-powered concentrators (estimated $7120 lower cost).

The ICER estimates (solar-powered O_2_ vs null case) were most sensitive to parameters related to the DALYs saved (eg, patient volume and mortality, Figure 2). The ICER is inversely proportional (*y *) to the DALYs saved and increases sharply as the denominator (DALYs saved) becomes small. Our findings suggest that solar-powered O_2_ would be most cost-effective (relative to no oxygen) in health facilities with high numbers of pneumonia cases and case fatality rate. In addition, solar-powered O_2_ would be cost-effective relative to grid-powered concentrators at facilities with unreliable grid electricity (>1.6% power outage, eFigure 1 in the Supplement). Overall, individual health facilities without prior oxygen that also have high patient volumes, acuity, and frequent power cuts may wish to invest in solar-powered O_2_. These characteristics are reflected across many African hospitals.^10,20,25^ On the other hand, our sensitivity analysis showed minimal change in ICER across variations in component prices of solar-powered O_2_ systems. These findings suggest that cost-effectiveness would be minimally threatened by fluctuations in component prices.

Analysis of the societal perspective suggests that costs incurred by patient families contribute 10% of the total costs associated with hypoxemic illnesses. Consideration of the costs borne by families is critical to an understanding of catastrophic household expenditures, which can propagate the cycle of poverty.^47^

### Limitations

Our study has several limitations. Our findings depend on the accuracy of the input parameters. Some parameters were based on few data (eg, the relative risk reduction in mortality with improved oxygen availability),^6^ and some were taken from our own experience implementing solar-powered O_2_ in Uganda.^18,19^ The ICER was sensitive to parameters that vary between health facilities, such as patient volume, case fatality rate, and consistency of grid electricity; therefore, our findings should be applied with caution to facilities that differ substantially from our base case assumptions. To mitigate this limitation, we used 1-way and multiway sensitivity analyses to describe the variation in the ICER with uncertainties in the inputs. Our model did not include contingencies such as surge demand (eg, respiratory virus outbreaks) and system failures (eg, solar-powered O_2_ battery depletion). These circumstances would be expected to increase the ICER through increased mortality (eg, insufficient oxygen supply) or costs (eg, backup cylinder oxygen). The choice of the comparator group would affect the ICER estimate. To provide several perspectives on the ICER, we used several comparators: null case with no oxygen (primary analysis), grid-powered oxygen concentrators, and fuel generator-powered concentrators (secondary analyses). Our DALY calculation did not include years lived with disability since children who survive an acute episode of hypoxemic severe pneumonia are expected to be discharged without permanent disability.^40,41^ The time horizon of our analysis was 10 years^20^; however, a longer time horizon could be more sensitive to variability in costs (eg, maintenance and equipment replacement costs) and stochastic events such as system failures and demand surges. Discounting of health outcomes is controversial.^20^ We used a 3% discount rate without age-weighting for our base case but provided a sensitivity analysis that included no discounting for health outcomes.^20^ The threshold used for cost-effectiveness in our study was based on GDP per capita; however, there has been some criticism of this methodology.^48^ Finally, whereas oxygen has utility for many clinical situations, our analysis focused specifically on oxygen therapy for inpatients younger than 5 years with hypoxemia. We therefore caution against extrapolating our findings to other clinical conditions. Our analysis is relevant to rural or remote hospitals in LMICs with a pediatric inpatient ward that can be served with a single oxygen concentrator and should not be applied to other settings. Additional details of the assumptions and limitations of the analysis can be found in the eMethods in the Supplement.

## Conclusions

The results of this economic evaluation suggest that solar-powered O_2_ is a cost-effective intervention relative to the null case (no oxygen) for treating children younger than 5 years with hypoxemia when compared with the GDP per capita of target LMICs. Solar-powered oxygen delivery also appears to be cost-effective relative to grid-powered concentrators and cost-saving relative to fuel generator-powered concentrators. Given the magnitude of pediatric pneumonia deaths, estimated at 900 000 per year,^2^ a life-saving and cost-effective intervention such as solar-powered O_2_ could represent an important tool toward improvements in global child survival.

## Notes

Supplement. **eAppendix.** Background Information **eMethods.** Additional Notes on Perspectives of Cost-Effectiveness Analysis **eTable 1.** Parameter Estimates for Direct Costs of Grid- and Fuel Generator-Powered Generators **eTable 2.** Health Effects and Costs Comparing Grid-Powered Concentrators and SPO_2_ **eFigure 1.** One-Way Sensitivity Analysis of ICER Estimate Comparing SPO_2_ to Grid-Powered Oxygen Concentrators **eFigure 2.** Probabilistic Multiway Sensitivity Analysis, Solar-Powered Oxygen Delivery (SPO_2_) Relative to Grid-Powered Concentrator **eTable 3.** Health Effects and Costs Comparing Fuel Generator-Powered Concentrators and SPO_2_ eReferences
