# Supplementary material for: Estimated Cost-effectiveness of Solar-Powered Oxygen Delivery for Pneumonia in Young Children in Low-Resource Settings
Source: JAMA Netw Open. 2021 Jun 24;4(6):e2114686. doi: 10.1001/jamanetworkopen.2021.14686 (PMC8226423; doi:10.1001/jamanetworkopen.2021.14686)
Supplement: Supplement. — eAppendix. Background Information eMethods. Additional Notes on Perspectives of Cost-Effectiveness Analysis eTable 1. Parameter Estimates for Direct Costs of Grid- and Fuel Generator-Powered Generators eTable 2. Health Effects and Costs Comparing Grid-Powered Concentrators and SPO2 eFigure 1. One-Way Sensitivity Analysis of ICER Estimate Comparing SPO2 to Grid-Powered Oxygen Concentrators eFigure 2. Probabilistic Multiway Sensitivity Analysis, Solar-Powered Oxygen Delivery (SPO2) Relative to Grid-Powered Concentrator eTable 3. Health Effects and Costs Comparing Fuel Generator-Powered Concentrators and SPO2 eReferences [file jamanetwopen-e2114686-s001.pdf]

# Supplementary Online Content

Huang Y, Mian Q, Conradi N, et al. Estimated cost-effectiveness of solar-powered oxygen delivery for pneumonia in young children in low-resource settings. *JAMA Netw Open*. 2021;4(6):e2114686. doi:10.1001/jamanetworkopen.2021.14686

**eAppendix.** Background Information

**eMethods.** Additional Notes on Perspectives of Cost-Effectiveness Analysis

**eTable 1.** Parameter Estimates for Direct Costs of Grid- and Fuel Generator-Powered Generators

**eTable 2.** Health Effects and Costs Comparing Grid-Powered Concentrators and $SPO_2$

**eFigure 1.** One-Way Sensitivity Analysis of ICER Estimate Comparing $SPO_2$ to Grid-Powered Oxygen Concentrators

**eFigure 2.** Probabilistic Multiway Sensitivity Analysis, Solar-Powered Oxygen Delivery ($SPO_2$) Relative to Grid-Powered Concentrator

**eTable 3.** Health Effects and Costs Comparing Fuel Generator-Powered Concentrators and $SPO_2$

**eReferences**

This supplementary material has been provided by the authors to give readers additional information about their work.

© 2021 Huang Y et al. *JAMA Network Open.*

**eAppendix.** Background Information

Our own group has experience in the implementation of SPO2 in Uganda, the Democratic Republic of the Congo, and Somalia. This experience informed the present cost-effectiveness analysis, both in terms of real-world costs of system components, as well as input parameters used to calculate health effects.

Proof-of-concept was demonstrated in a pilot study of 28 patients in Jinja, Uganda.[1] We next conducted a randomized controlled non-inferiority trial comparing SPO2 to cylinder oxygen in children under 13 years of age hospitalized with hypoxemia at two hospitals in Uganda.[2] The study found that SPO2 was safe and non-inferior to cylinder oxygen with respect to duration of hospitalization (primary outcome) as well as mortality, duration of hypoxemia, and time to recovery of hypoxemia, tachypnea, and tachycardia (secondary outcomes).[2] We are currently conducting a multi-center stepped wedge cluster randomized controlled trial of SPO2 at 20 health facilities across Uganda examining the mortality benefit of SPO2.[3]

© 2021 Huang Y et al. *JAMA Network Open.*

**eMethods.** Additional Notes on Perspectives of Cost-Effectiveness Analysis

The health sector represents a government-funded public health sector (e.g., the Ministry of Health of Uganda) which would be responsible for implementing and maintaining SPO2 systems. The healthcare sector perspective was included in the cost-effectiveness analysis because it informs the expenditures that would be necessary for a hospital or health system to implement SPO2, which is relevant for budgetary impacts. The societal perspective expands upon the healthcare perspective by adding costs that patient families experience when their child is admitted to hospital, and is the recommended perspective in WHO CHOICE.[4]

*One-way sensitivity analysis*

Starting with the base case assumptions, the input parameters were varied one at a time within the range of plausible values (Table 1). All other variables were held constant at the base case values. The ICER was calculated at each extremum of the range, and was plotted on a tornado plot (Figure 1). The circles represent the base case and the whiskers represent the upper and lower limits of the ICER, at each extreme of the input parameter.

For selected input parameters, we explored the relationship between the parameter value and the ICER by varying the parameter over its plausible range while holding the other inputs constant at their base case values. A graphical representation was used to display the independent effects of key inputs (Figure 2).

© 2021 Huang Y et al. *JAMA Network Open.*

*Multi-way sensitivity analysis*

In computing the ICER, there is uncertainty in both the incremental costs (numerator) and health effects (denominator). These were displayed in a plane in which each point was an estimate of the ICER, based on a probabilistic sampling of the input parameters.[5] The cost-effectiveness threshold (GDP *per capita*) was plotted on the same plane, defining a space in which the ICER was favourable (Figure 3A). Next, the individual ICER estimates were used to construct a cost-effectiveness acceptability curve. The curve was constructed by varying the threshold value of the ICER and determining the posterior probability of the ICER meeting that threshold.[5] As the threshold value rises, the probability of the ICER being judged cost-effective increases.[5] The willingness-to-pay thresholds were displayed on the x-axis and the probability that SPO2 has an ICER (relative to the null case) below this threshold was plotted on the y-axis (Figure 3B).

*Model assumptions and justifications*

1. *Input parameters.* Numerous assumptions for input parameters (base case and plausible range) are summarized in Table 1. Where possible, these were taken from the literature for maximum generalizability. Where we could not find published data, we used parameters or costs based on our own experience implementing SPO2 in Uganda.[2] For sensitivity analyses, the parameters were assumed to be random variables with a Poisson, beta, or gamma probability distribution functions for frequencies, proportions, and costs or time periods, respectively (Table 1).

© 2021 Huang Y et al. *JAMA Network Open.*

2. *Model simplifications.* We did not include effects of surges in demand (e.g., respiratory virus outbreak) or system failures (e.g., battery depletion) that could affect the cost-effectiveness of SPO2. In our experience, these are low-probability events[2] and were not included in our cost-effectiveness model.

3. *Comparator group.* The comparator group was assumed to be the null case (no oxygen) for our primary analysis. This was based on the WHO-CHOICE methodology.[4] Ideally, the comparator group should represent the standard of care.[5] Medical oxygen is not available on the pediatric wards of many resource-limited hospitals,[6,7] justifying this comparator group. In addition to this primary analysis, we also conducted secondary analyses comparing SPO2 to grid- and fuel generator-powered concentrators since these are alternative methods of oxygen delivery.

4. *DALY calculation.* For the DALY calculation, we neglected the YLD, such that YLL accounted for all the DALYs lost. This was based on the assumption that children who recover from pneumonia do not have residual morbidity. Two previous studies examined the long-term outcomes in hospitalized children with pneumonia and found a small increase in restrictive lung disease.[8,9] However, given that these studies were not conducted in low-resource settings, did not clearly show that oxygen treatment was associated with subsequent pathogenesis, and did not control for environmental factors contributing to future respiratory disease, we opted to focus on mortality and forgo disability weighting among survivors.

5. *Time horizon.* Following the recommendations of WHO-CHOICE as a standard approach to cost-effectiveness analysis,[10] we assumed a time horizon of 10 years.

© 2021 Huang Y et al. *JAMA Network Open.*

6. *Discounting and age-weighting of health effects.* Discounting of health effects was used in the base case (discount rate 3%). This assumes a preference for present health over future years of good health. Age-weighting of DALYs was not used, as this is controversial.[10]

7. *Threshold for cost-effectiveness*. The threshold below which the ICER must be for an intervention to be considered cost-effective was assumed to be the GDP *per capita*. Other cost-effectiveness studies in high-income settings have used $50 000 per DALY saved, based on renal dialysis in the USA, because there is general (political) agreement that there is willingness to pay for renal dialysis.[5] The argument that the cost-effectiveness threshold varies in poorer countries is debatable.[5] We began with the GDP *per capita* of Uganda, where SPO2 was pioneered. For maximum stringency, we also used the lowest GDP *per capita* in the world (South Sudan, GDP of $220) to evaluate the cost-effectiveness of SPO2 (*vs* no oxygen). Of note, despite the usefulness of a benchmark for comparison, there is no scientific justification for the GDP or any other cost-effectiveness threshold over another.

8. *Use of SPO2 restricted to severe childhood pneumonia with hypoxemia at a rural or remote pediatric ward in a LMIC*. The disease and patient demographic of interest was hypoxemia in children <5 years of age. Childhood pneumonia is the most common cause for hypoxemia and is a leading cause of death globally.[11] Our analysis of SPO2 cost-effectiveness was restricted to this condition and should not be extrapolated to other patient groups or clinical conditions for which the health effects may be different. Furthermore, we assumed that SPO2 would be used in a pediatrics ward in a LMIC hospital in a remote or rural location (where grid power is unreliable and access to cylinder oxygen is challenging). In this setting, a single oxygen concentrator (5L/min, two patients at a time) is generally sufficient.

© 2021 Huang Y et al. *JAMA Network Open.*

**eTable 1.** Parameter Estimates for Direct Costs of Grid- and Fuel Generator-Powered Generators

| Parameter | Base | Range | Distribution | Reference |
|---|---|---|---|---|
| *Cost of grid electricity* | | | | |
| **Price of grid electricity in Uganda (USD/kWh)** | 0.16 | 0.09 – 0.23 | Gamma | (Meyer et al., 2015)[12] |
| **Proportion of time without grid power** | 0.07 | 0.01 – 0.58 | Beta | (Otiangala et al. 2020)[13] |
| *Cost of fuel-powered electrical generator* | | | | |
| **Volume of gasoline required by fuel generator (L/kWh)** | 0.57 | 0.25 – 1.38 | Gamma | (Muslim et al., 2018; Farquharson et al., 2018)[14,15] |
| **Price of gasoline in Uganda (USD/L)** | 1.17 | 0.94 – 1.40 | Gamma | (The World Bank, 2016)[16] |
| **Price of gasoline generator unit (USD)** | 400 | 200 – 1,000 | Gamma | (Muslim et al., 2018; Babatunde et al., 2019)[14,17] |
| **Life span of gasoline generator (years)** | 5 | 4 – 10 | Gamma | (Muslim et al., 2018; Babatunde et al., 2019)[14,17] |
| **Cost of gasoline generator maintenance over 10 years** | 1,000 | 800 – 3,320 | Gamma | (Muslim et al., 2018; Babatunde et al., 2019)[14,17] |

© 2021 Huang Y et al. *JAMA Network Open.*

**eTable 2.** Health Effects and Costs Comparing Grid-Powered Concentrators and $SPO_2$

| Parameter | Grid-powered concentrator (95%CI) | SPO2 (95%CI) | Prevented by SPO2 (95%CI) | Difference (%) (95%CI) |
|---|---|---|---|---|
| ***Events*** | | | | |
| Hospitalizations with hypoxemia | 869<br>(78-3 580) | 869<br>(78-3 580) | 0 | 0 |
| Deaths | 80<br>(6-371) | 77<br>(6-352) | 3<br>(0-30) | 4<br>(0-22) |
| DALYs | 21 595<br>(2 586-134 057) | 21 675<br>(2 586-134 520) | 80<br>(0-1 098) | 0<br>(0-3) |
| ***Costs (USD)*** | | | | |
| *Direct medical costs* | | | **Additional cost SPO2 (95%CI)** | |
| SPO2 (capital and maintenance) | 8 446<br>(4 141-14 354) | 22 939<br>(15 034-33 999) | 14 493<br>(9 005-22 984) | 170<br>(90 to 390) |
| Consumables (grid power cost) | 3 329<br>(168-14 454) | 0 | 3 329<br>(168-14 454) | - |
| Antibiotics and other treatment | 138 407<br>(11 650-518 564) | 138 407<br>(11 650-518 564) | 0 | 0 |
| **Total medical costs** | 150 181<br>(18 913-540 620) | 161 346<br>(31 913-543 164) | 11 165<br>(-602 to 20 928) | 7<br>(0-77) |
| *Nonmedical costs* | | | | |
| Loss of earnings by caregiver | 4 594<br>(278-20 166) | 4 603<br>(278-20 246) | 9<br>(-21 to 109) | 0<br>(-1 to 2) |
| Other direct nonmedical | 13 673<br>(519-73 499) | 13 689<br>(523-73 553) | 17<br>(-36 to 211) | 0<br>(0 to 2) |
| **Total nonmedical costs** | 18 267<br>(950-91 296) | 18 293<br>(954-91 594) | 25<br>(-56 to 317) | 0<br>(-1 to 2) |
| **Total cost** | 168 449<br>(20 485-610 863) | 179 639<br>(33 669-614 795) | 11 190<br>(-532 to 20 961) | 7<br>(0-71) |

© 2021 Huang Y et al. *JAMA Network Open.*

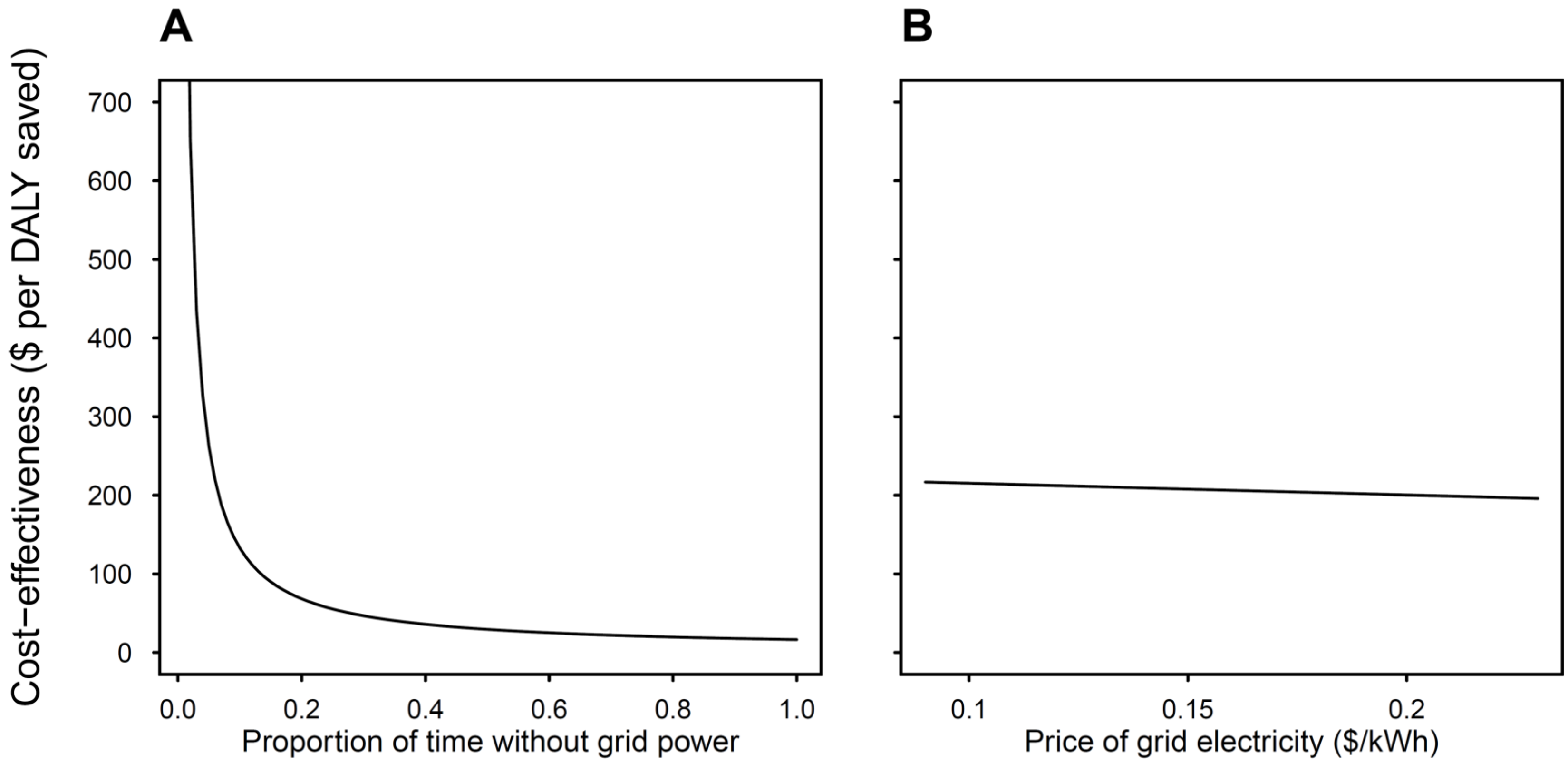


**eFigure 1.** One-Way Sensitivity Analysis of ICER Estimate Comparing $SPO_2$ to Grid-Powered Oxygen Concentrators

**A.** The ICER estimate was sensitive to changes in the proportion of time without grid power, following an inverse relationship. ICER rose steeply when grid electricity was reliable. The ICER was favorable (<$604 per DALY saved) as long as the proportion of time without grid power was >1.6%. **B.** The ICER was relatively insensitive to variations in the price of grid electricity.

© 2021 Huang Y et al. *JAMA Network Open.*

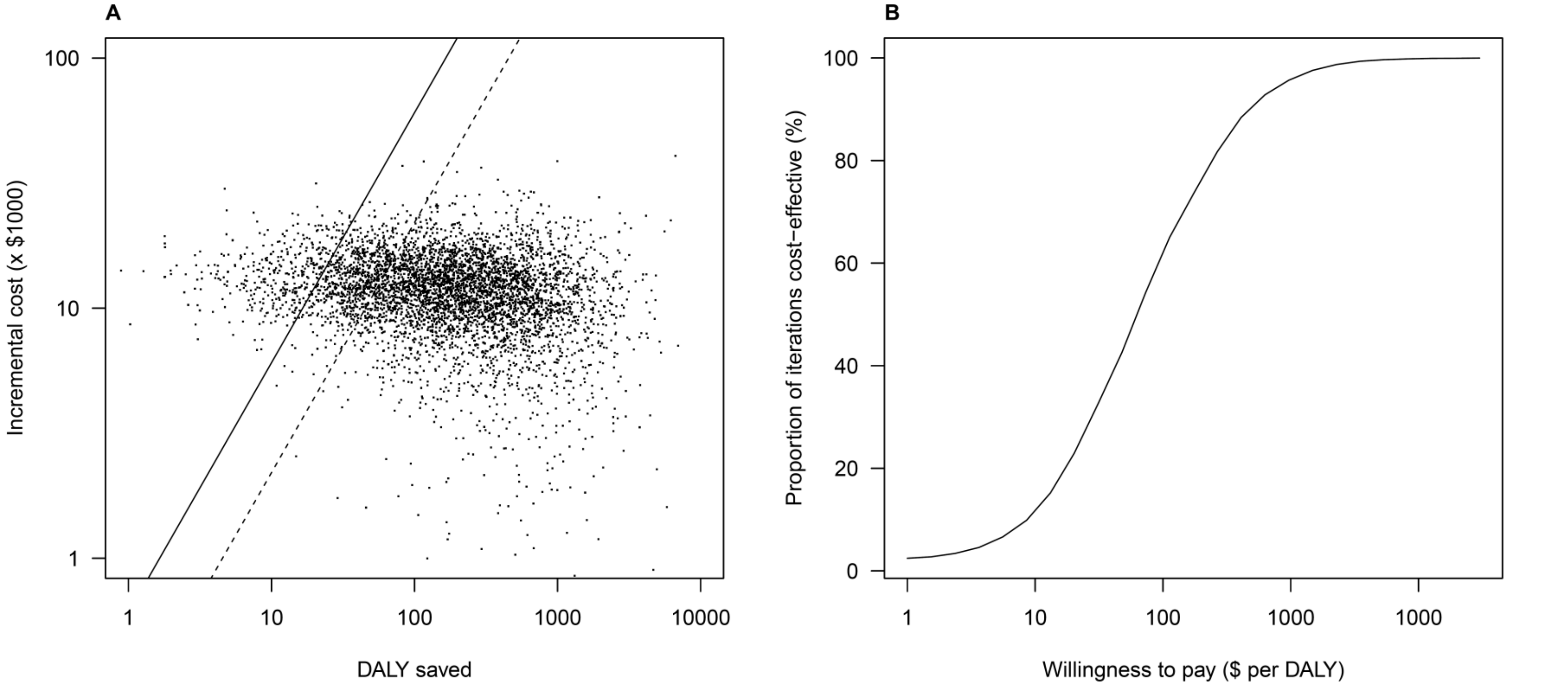


**eFigure 2.** Probabilistic Multi-Way Sensitivity Analysis, Solar-Powered Oxygen Delivery ($SPO_2$) Relative to Grid-Powered Concentrator

**A.** Scatterplot showing the costs and DALYs saved in 5 000 simulations. The solid line represents a cost-effectiveness threshold of $604/DALY saved (GDP of Uganda) and the dashed line shows a threshold of $220/DALY saved (GDP of South Sudan). 92.4% and 78.2% of simulations were cost-effective using these two thresholds, respectively. **B.** Cost-effectiveness acceptability curve: we can be 95% confident that SPO2 will be cost-effective beyond a willingness-to-pay threshold of $849/DALY saved.

© 2021 Huang Y et al. *JAMA Network Open.*

**eTable 3.** Health Effects and Costs Comparing Fuel Generator-Powered Concentrators and $SPO_2$

| **Parameter** | **Fuel powered concentrator (95%CI)** | **SPO2 (95%CI)** | **Prevented by SPO2 (95%CI)** | **Difference (%) (95%CI)** |
|---|---|---|---|---|
| ***Events*** | | | | |
| Hospitalizations with hypoxemia | 869 (78-3 580) | 869 (78-3 580) | 0 | 0 |
| Deaths | 77 (6-352) | 77 (6-352) | 0 | 0 |
| DALYs | 21 675 (2 586-134 520) | 21 675 (2 586-134 520) | 0 | 0 |
| ***Costs (USD)*** | | | | |
| *Direct medical costs* | | | **Additional cost SPO2 (95%CI)** | |
| SPO2 (capital and maintenance) | 15 794 (8 950-26 088) | 22 939 (15 034-33 999) | 7 145 (-1 222 to 16 272) | 45 (-6 to 140) |
| Consumables (fuel) | 14 265 (657-65 869) | 0 | -14 265 (-65 869 to -657) | |
| Antibiotics and other treatment | 138 407 (11 650-518 564) | 138 407 (11 650-518 564) | 0 | 0 |
| **Total medical costs** | 168 466 (25 982-587 353) | 161 346 (31 913-543 164) | -7 120 (-59 876 to 11 673) | -4 (-17 to 31) |
| *Nonmedical costs* | | | | |
| Loss of earnings by caregiver | 4 603 (278-20 246) | 4 603 (278-20 246) | 0 | 0 |
| Other direct nonmedical | 13 689 (523-73 553) | 13 689 (523-73 553) | 0 | 0 |
| **Total nonmedical costs** | 18 293 (954-91 594) | 18 293 (954-91 594) | 0 | 0 |
| **Total cost** | 186 759 (27 428-661 893) | 179 639 (33 669-614 795) | -7 120 (-59 876 to 11 673) | -4 (-15 to 28) |

© 2021 Huang Y et al. *JAMA Network Open.*

**eReferences**


1. Turnbull H, Conroy A, Opoka R, et al. Solar-powered oxygen delivery: proof of concept. 2016;20(5):696-703.
2. Hawkes M, Conroy A, Namasopo S, et al. Solar-powered oxygen delivery in low-resource settings: a randomized clinical noninferiority trial. *JAMA Pediatr.* 2018;172(7):694-696.
3. Conradi N, Mian Q, Namasopo S, et al. Solar-powered oxygen delivery for the treatment of children with hypoxemia: protocol for a cluster-randomized stepped-wedge controlled trial in Uganda. *Trials.* 2019;20(1):679.
4. WHO. World Health Organization. Choosing interventions that are cost effective (WHO-CHOICE). Available at: https://www.who.int/choice/cost-effectiveness/en/. Accessed 13 Feb 2021. In:2010.
5. Weintraub WS, Cohen DJ. The limits of cost-effectiveness analysis. *Circ Cardiovasc Qual Outcomes.* 2009;2(1):55-58.
6. Nabwire J, Namasopo S, Hawkes M. Oxygen availability and nursing capacity for oxygen therapy in Ugandan paediatric wards. *J Trop Pediatr.* 2018;64(2):97-103.
7. Otiangala D, Agai NO, Olayo B, et al. Oxygen insecurity and mortality in resource-constrained healthcare facilities in rural Kenya. *Pediatric Pulmonology.* 2020.
8. Grimwood K, Chang AB. Long-term effects of pneumonia in young children. *Pneumonia.* 2015;6(1):101-114.
9. Edmond K, Scott S, Korczak V, et al. Long term sequelae from childhood pneumonia; systematic review and meta-analysis. *PloS one.* 2012;7(2):e31239-e31239.
10. WHO. Methods and data sources for global burden of disease estimates 2000-2011. Available at: https://www.who.int/healthinfo/statistics/GlobalDALYmethods_2000_2011.pdf. Accessed 13 Feb 2021.
11. Subhi R, Adamson M, Campbell H, et al. The prevalence of hypoxaemia among ill children in developing countries: a systematic review. *Lancet Infect Dis.* 2009;9(4):219-227.
12. Meyer R, Tenenbaum B, Hosier R. Promoting Solar Energy through Auctions. 2015.
13. Otiangala D, Agai NO, Olayo B, et al. Oxygen insecurity and mortality in resource-constrained healthcare facilities in rural Kenya. *Pediatr Pulmonol.* 2020;55(4):1043-1049.
14. Muslim H, Alkhazraji A. Feasibility study of using 2kWp residential PV system comparing with 2.5kVA gasoline generator (Case study: Baghdad city). *International Journal of Energy and Environment (IJEE).* 2018.
15. Farquharson D, Jaramillo P, Samaras C. Sustainability implications of electricity outages in sub-Saharan Africa. *Nature Sustainability.* 2018;1(10):589-597.
16. Bank TW. Pump price for gasoline (US$ per liter) - Uganda. 2016.
17. Babatunde OM, Munda JL, Hamam Y. Selection of a Hybrid Renewable Energy Systems for a Low-Income Household. *Sustainability.* 2019;11(16):4282.


© 2021 Huang Y et al. *JAMA Network Open.*
